# Attenuated LKB1-SIK1 signaling promotes epithelial-mesenchymal transition and radioresistance of non–small cell lung cancer cells

**DOI:** 10.1186/s40880-016-0113-3

**Published:** 2016-06-07

**Authors:** Yuan-Hu Yao, Yan Cui, Xiang-Nan Qiu, Long-Zhen Zhang, Wei Zhang, Hao Li, Jin-Ming Yu

**Affiliations:** Shandong University School of Medicine, Jinan, 250012 Shandong P. R. China; Department of Radiation Oncology, The Affiliated Hospital of Xuzhou Medical University, Xuzhou, 221002 Jiangsu P. R. China; Leibniz Institute on Aging-Fritz Lipmann Institute, Jena, 07745 Germany; Department of Radiation Oncology, Jinling Hospital, Medical School of Nanjing University, Nanjing, 210002 Jiangsu P. R. China; Jiangsu Key Laboratory of Biological Therapies for Tumors, Xuzhou Medical University, Xuzhou, 221002 Jiangsu P. R. China; Department of Radiation Oncology, Shandong Cancer Hospital and Institute, Shandong University, Jinan, 250117 Shandong P. R. China

**Keywords:** Radioresistance, Lung cancer, Metastasis, Epithelial-mesenchymal transition, LKB1, SIK1

## Abstract

**Background:**

Radiotherapy is one of the main therapeutic approaches for non–small cell lung cancer (NSCLC). However, radioresistant cancer cells can eventually cause tumor relapse and even fatal metastasis. It is thought that radioresistance and metastasis could be potentially linked by epithelial-mesenchymal transition (EMT). In this study, we established radioresistant NSCLC cells to investigate the potential relationship among radioresistance, EMT, and enhanced metastatic potential and the underlying mechanism involving liver kinase B1 (LKB1)-Salt-inducible kinase 1 (SIK1) signaling.

**Methods:**

The radioresistant cell lines A549R and H1299R were generated by dose-gradient irradiation of the parental A549 and H1299 cells. The radioresistance/sensitivity was evaluated by Cell Counting Kit-8 assay, apoptosis analysis, and/or clonogenic cell survival assay. The EMT phenotype and the signaling change were assessed by Western blotting. The abilities of invasion and migration were evaluated by transwell assays and wound healing assays.

**Results:**

The radioresistant cell lines A549R and H1299R displayed mesenchymal features with enhanced invasion and migration. Mechanistically, A549R and H1299R cells had attenuated LKB1-SIK1 signaling, which leaded to the up-regulation of Zinc-finger E-box-binding homeobox factor 1 (ZEB1)—a transcription factor that drives EMT. Re-expression of LKB1 in A549R cells reversed the EMT phenotype, whereas knockdown of LKB1 in H1299R cells further promoted the EMT phenotype. Moreover, re-expression of *LKB1* in A549 cells increased the radiosensitivity, whereas knockdown of *LKB1* in H1299 cells decreased the radiosensitivity.

**Conclusions:**

Our findings suggest that attenuated LKB1-SIK1 signaling promotes EMT and radioresistance of NSCLC cells, which subsequently contributes to the enhanced metastatic potential. Targeting the LKB1-SIK1-ZEB1 pathway to suppress EMT might provide therapeutic benefits.

## Background

Lung cancer is a leading cause of cancer-related death worldwide [1] and in China [2]. According to a statistic of the major cancers in China, 2011, lung cancer had the highest incidence in males and the second highest incidence in females, and it accounted for 27.08% and 25.08% of all cancer deaths in males and females, respectively [2].

Radiotherapy is a common treatment of lung cancer; despite advances in radiation technology, its efficacy is limited, and the prognosis of patients with lung cancer remains poor [3]. After definitive radiotherapy, up to one-third of patients will have local recurrence and distant metastasis [3]. Radioresistant cancer cells likely play a crucial role in the recurrence and metastasis of lung cancer after radiotherapy. Several studies suggested that radioresistant cancer cells have enhanced invasion and metastasis potential [4, 5].

Epithelial-mesenchymal transition (EMT) is a complex process accompanied by loss of epithelial markers such as E-cadherin and acquisition of mesenchymal markers such as vimentin and fibronectin [6]. During EMT, epithelial cells lose cell-cell contacts and apical-basal polarity and acquire migratory properties [7]. Emerging evidence suggests that EMT is a key step toward cancer metastasis and is associated with radioresistance [8]. It has been reported that in vitro cultured lung cancer cells that survived ionizing radiation treatment display the EMT phenotype and have increased invasion ability [9]. More importantly, it has also been observed that radiotherapy may induce EMT in vivo, demonstrated by comparing surgically resected non–small cell lung cancer (NSCLC) specimens before and after radiotherapy [10].

Liver kinase B1 (LKB1), also known as serine/threonine protein kinase 11 (STK11), functions in many types of cancer as a tumor suppressor. Particularly, *LKB1* is the third most commonly mutated gene in lung adenocarcinoma [11]. Retrospective studies of patient cohorts suggest that LKB1 expression is negatively associated with lymph node metastasis [12, 13]. Using the mouse model of oncogenic Kras-driven lung cancer, LKB1 has been shown to be a critical barrier to lung cancer initiation and metastasis [14]. LKB1 directly phosphorylates and activates 5′-adenosine monophosphate-activated protein kinase (AMPK) and AMPK-related kinases to control cell metabolism, proliferation, and polarity, which at least partly accounts for its tumor suppressor function [15, 16]. Salt-inducible kinase 1 (SIK1) is a member of the AMPK-related kinase family and is also a critical effector of LKB1 to suppress metastasis [17]. It has been shown that LKB1-SIK1 signaling suppresses EMT by repressing the expression of several transcriptional factors critically involved in EMT, including snail2, twist, and Zinc-finger E-box-binding homeobox factor 1 (ZEB1) [18].

In this study, we established radioresistant NSCLC cells lines A549R and H1299R and investigated the potential relationship among radioresistance, EMT, and enhanced metastatic potential and the underlying mechanism involving LKB1-SIK1 signaling.

## Methods

### Cell lines and culture conditions

Human lung cancer cell lines A549 and H1299 were purchased from Keygen Biotech (Nanjing, China). The radioresistant derivatives A549R and H1299R were generated by dose-gradient irradiation of the parental cells. All cells were maintained in RPMI-1640 medium (Gibco, New York, MD, USA) containing 10% fetal bovine serum at 37°C with 5% CO_2_ in a humidified incubator.

### Dose-gradient irradiation

Irradiation was performed at a dose rate of 300 cGy/min at room temperature using a Varian 23 EX Clinac linear accelerator (Varian Medical Systems, Inc., Palo Alto, CA, USA). For the first irradiation, A549 and H1299 cells were grown to 60%–70% confluence and irradiated with 2 Gy of X-ray; the culture medium was replenished immediately after irradiation. When the cells reached the confluence of more than 80%, they were trypsinized and passaged. After two passages, the same irradiation and cell propagation procedure was performed. The procedure was further repeated with gradually increased radiation dose, and each dose was used twice. In total, the cells received 60 Gy of radiation (2 × 2 Gy, 2 × 4 Gy, 2 × 6 Gy, 2 × 8 Gy, and 2 × 10 Gy). The surviving cells were propagated and passaged for five or more generations before being used for other experiments.

### Cell viability/proliferation assay with Cell Counting Kit-8

A Cell Counting Kit-8 (CCK-8) kit (Dojindo Laboratories, Kumamoto, Japan) was used to determine cell viability and proliferation after irradiation. Briefly, the cells were seeded in a 96-well plate (3000 cells/well, four replicates for each cell line) and incubated overnight. The cells were irradiated with five different doses (0, 2, 4, 6, and 8 Gy) and then incubated for further 48 h. The cells were replenished with a medium containing CCK-8 solution (10 μL CCK-8 in 100 μL medium) and incubated for another 2 h; then the absorbance at 450 nm was measured using a microplate reader (Bio-Tek Instruments, Winooski, VT, USA). The survival rate of cells was calculated as the normalized absorbance to the non-irradiated controls.

### Apoptosis detection

Cells were stained with an Annexin V-FITC detection kit (KeyGen, Nanjing, Jiangsu, China), following the manufacturer’s instructions, and analyzed with a BD FACScan system (BD Biosciences, San Jose, CA, USA). The graph was plotted using Flowjo 7.6.5 software (FLOWJO LLC, Ashland, KY, USA).

### Plasmids and transfections

The pEGFP-LKB1, pEGFP-Ctrl, pshLKB1, and pshCtrl plasmids were constructed by GenePharma (Shanghai, China). Human LKB1 open reading frame was inserted in-frame with enhanced green fluorescent protein (EGFP) into the pEGFP-N1 vector to obtain the pEGFP-LKB1 vector. pGenesil-1 is a derivative of the pEGFP-C1 vector, which contains a human U6 promoter to drive short hairpin RNA (shRNA) expression. A DNA fragment encoding an shRNA against human LKB1 was inserted into the pGenesil-1 vector to obtain pshLKB1; the scrambled shRNA was also cloned into pGenesil-1 to obtain pshCtrl. The target sequence of LKB1 was 5′-GGTACTTCTGTCAGCTGATTG-3′, and the scrambled shRNA sequence was 5′-GTTCTCCGAACGTGTCACGTT-3′. Transient transfection was performed with Lipofectamine 2000 (Invitrogen, Shanghai, China), following the manufacturer’s instructions. Twenty-four hours after transfection, the cells were harvested for either Western blot analysis or further functional tests.

### Western blotting and antibodies

Western blotting was performed as described previously [19]. The following primary antibodies were used: LKB1 (ab15095, 1:100) from Abcam (Cambridge, UK); E-cadherin (BS1097, 1:500), vimentin (BS1776, 1:500), β-actin (BS6007 M, 1:10,000), p-CHK2 (p-T68) (BS4043, 1:500), and γ-H2AX (p-S139) (BS4760, 1:500) from Bioworld Technology (Nanjing, Jiangsu, China); and SIK1 (51045-1-AP, 1:1000) and ZEB1 (21544-1-AP, 1:1000) from Proteintech Group (Wuhan, Hubei, China). All primary antibodies were incubated with the blot at 4°C overnight. The signals were detected with an Odyssey Infrared Imaging system (LI-COR, Lincoln, NE, USA). For quantification of the protein levels, the intensity of each strip was analyzed by Image J software (NIH, Bethesda, MD, USA). The average intensities of the proteins were normalized to β-actin. The relative protein levels are presented as mean ± standard deviation (SD).

### Cell invasion assay

Invasion ability of the cells was determined using a modified two-chamber plate with a pore size of 8 μm. The transwell filter inserts were coated with Matrigel (BD Biosciences, New York, NJ, USA), and 5 × 10^4^ cells were seeded in serum-free medium in the upper chamber. After incubation for 48 h at 37°C, cells in the upper chamber were carefully removed with a cotton swab, and the cells that had traversed the membrane were fixed in methanol and stained with crystal violet. The cells were counted under an inverted microscope and photographed. If transient transfection was performed prior to the assay, the cells were seeded 24 h after the transfection.

### Cell migration assay

Migration assay was performed similarly to the invasion assay, with the following differences: the transwell filter inserts were not coated; 3 × 10^4^ cells were seeded; and the incubation time was 24 h before fixation.

### Wound healing assay

A wound was made by dragging a yellow pipette tip along the center of the plate. The distance between the cells bordering the wound was measured after 24 h. Images were taken with a digital camera under a phase-contrast microscope. If transient transfection was performed prior to the assay, the cells were wounded 24 h after the transfection.

### Clonogenic cell survival assay

The cells were seeded in six-well plates (200, 400, 1000, 3000, and 5000 cells/well in triplicate, corresponding to the radiation dose of 0, 2, 4, 6, and 8 Gy, respectively). After overnight incubation, the cells were irradiated with the respective dose. The medium was replenished after the irradiation, and the cells were cultured for 14 days prior to formaldehyde fixation and crystal violet staining. The colonies with 50 or more cells were counted. The plating efficiency was calculated as the ratio of the colonies number to the plated cell number. The survival fraction was calculated as the normalized plating efficiency to the non-irradiated controls. Using GraphPad Prism 5.0 software (GraphPad Software, La Jolla, CA, USA), the survival curves were fitted to a linear-quadratic model to estimate the sensitizer enhancement ratio.

### Statistical analysis

All statistical analyses were performed by using SPSS version 16.0 software (IBM, Chicago, IL, USA) except for those specially stated. All values are shown as mean ± SD. Student’s *t* test and one-way analysis of variance (ANOVA) were used to evaluate significance. *P* values less than 0.05 were considered statistically significant. All tests were two-tailed.

## Results

### Radioresistant A549R and H1299R cells displayed the EMT phenotype

**Fig. 1:**
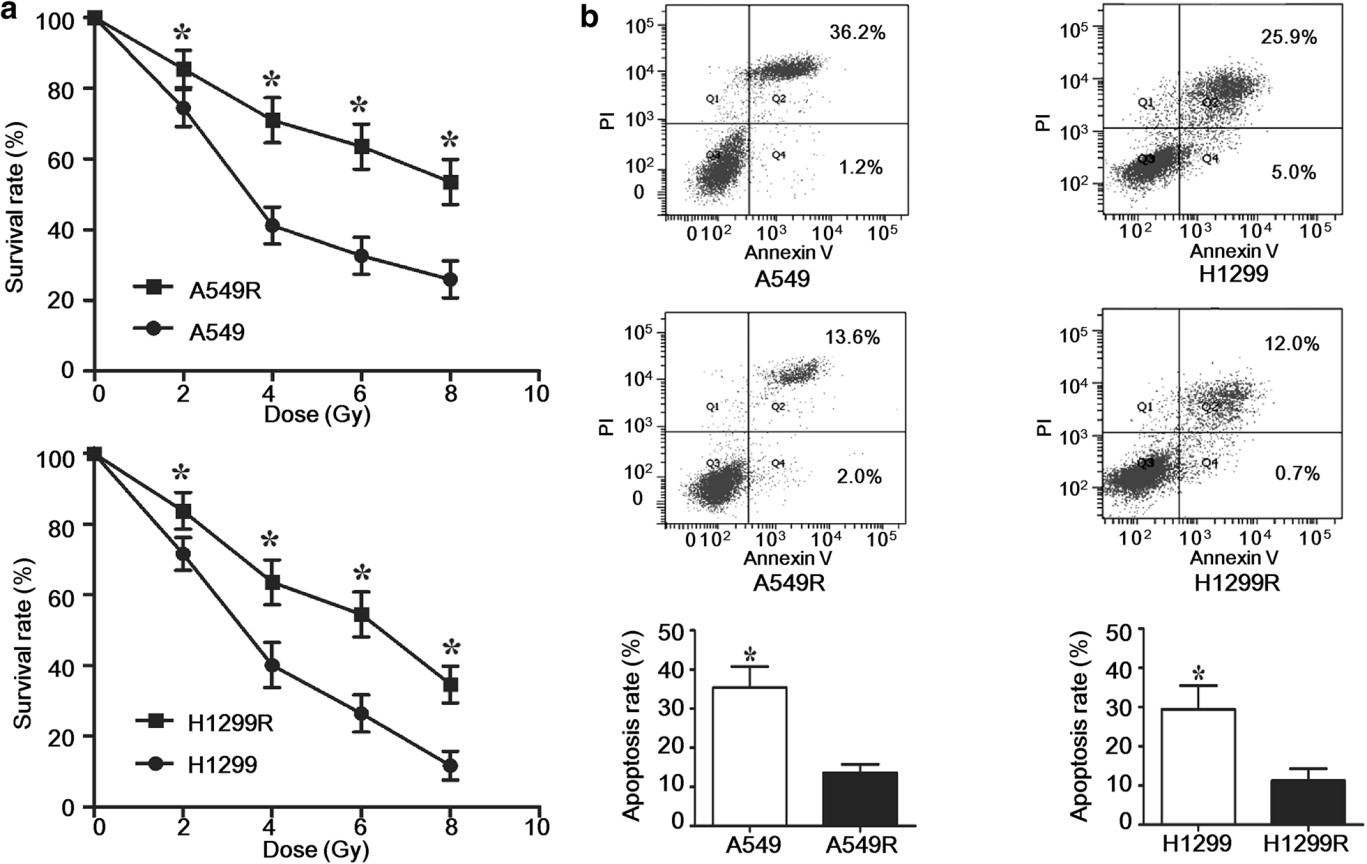
A549R and H1299R cells were more resistant to ionizing radiation than the parental A549 and H1299 cells. **a** The cells were irradiated with the indicated dose; 48 h later, Cell Counting Kit-8 assay was performed to detect the viability and proliferation of the cells. The survival rate was normalized to the non-irradiated controls. *P < 0.05 (Student’s *t* test). **b** The cells were irradiated with 2 Gy of X-ray; 24 h later, the apoptosis of the cells was determined by Annexin V-FITC/propidium iodide (PI) staining and fluorescence-activated cell sorting analysis. Representative results are shown. The apoptotic fraction (Q2 + Q4) from three independent experiments was used to plot the *bar chart*. *P < 0.05 (Student’s *t* test)

**Fig. 2:**
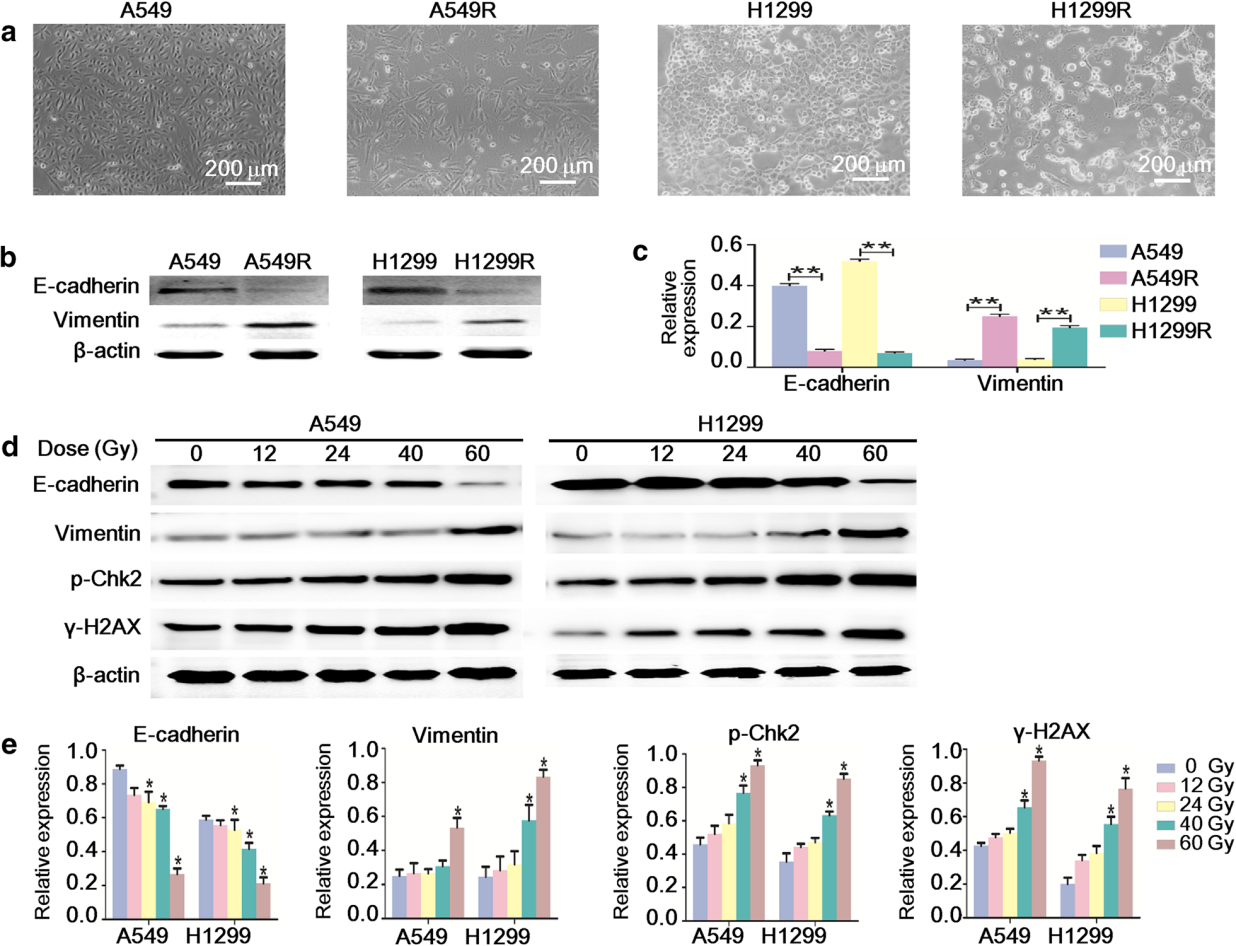
The radioresistant non-small cell lung cancer cells displayed the epithelial–mesenchymal transition phenotype. **a** The cells were photographed by light microscope at ×100 magnification. **b** Western blot analysis of E-cadherin and vimentin expression in A549, A549R, H1299, and H1299R cells. **c** The quantification of the protein levels in **b** **P < 0.01 (Student’s *t* test). **d** Western blot analysis of the dynamic change of the protein levels of E-cadherin, vimentin, phosphor-Chk2 (p-Chk2), and γ-H2AX during the accumulation of the radiation dose. **e** The quantification of the protein levels in **d**. *P < 0.05 (ANOVA-SNK-q′) versus the respective 0-Gy group

By dose-gradient irradiation of the parental A549 and H1299 cells to the total dose of 60 Gy, we derived the respective radioresistant cell lines A549R and H1299R. Compared with the parental cells, A549R and H1299R demonstrated a significantly increased survival rate and reduced apoptosis after ionizing radiation (Fig. 1), supporting the hypothesis that these cells were more resistant to radiation. A549R and H1299R cells showed enlarged size and mesenchymal morphology indicative of EMT (Fig. 2a). Examination of the epithelial marker E-cadherin and the mesenchymal marker vimentin by Western blotting confirmed dramatic down-regulation of E-cadherin and concomitant up-regulation of vimentin in A549R and H1299R cells (Fig. 2b, c). These observations suggest that A549R and H1299R cells underwent EMT. EMT was likely induced gradually during the dose-gradient irradiation procedure. Therefore, we examined the dynamic change of E-cadherin and vimentin expression. Indeed, only when the radiation dose was accumulated to 60 Gy was the expression level of E-cadherin and vimentin significantly changed (Fig. 2d, e). The DNA damage response was also gradually induced during the accumulation of radiation, indicated by the increased phosphorylation of Chk2 (p-T68) and γ-H2AX (Fig. 2d, e).

### A549R and H1299R cells showed enhanced ability of invasion and migration

**Fig. 3:**
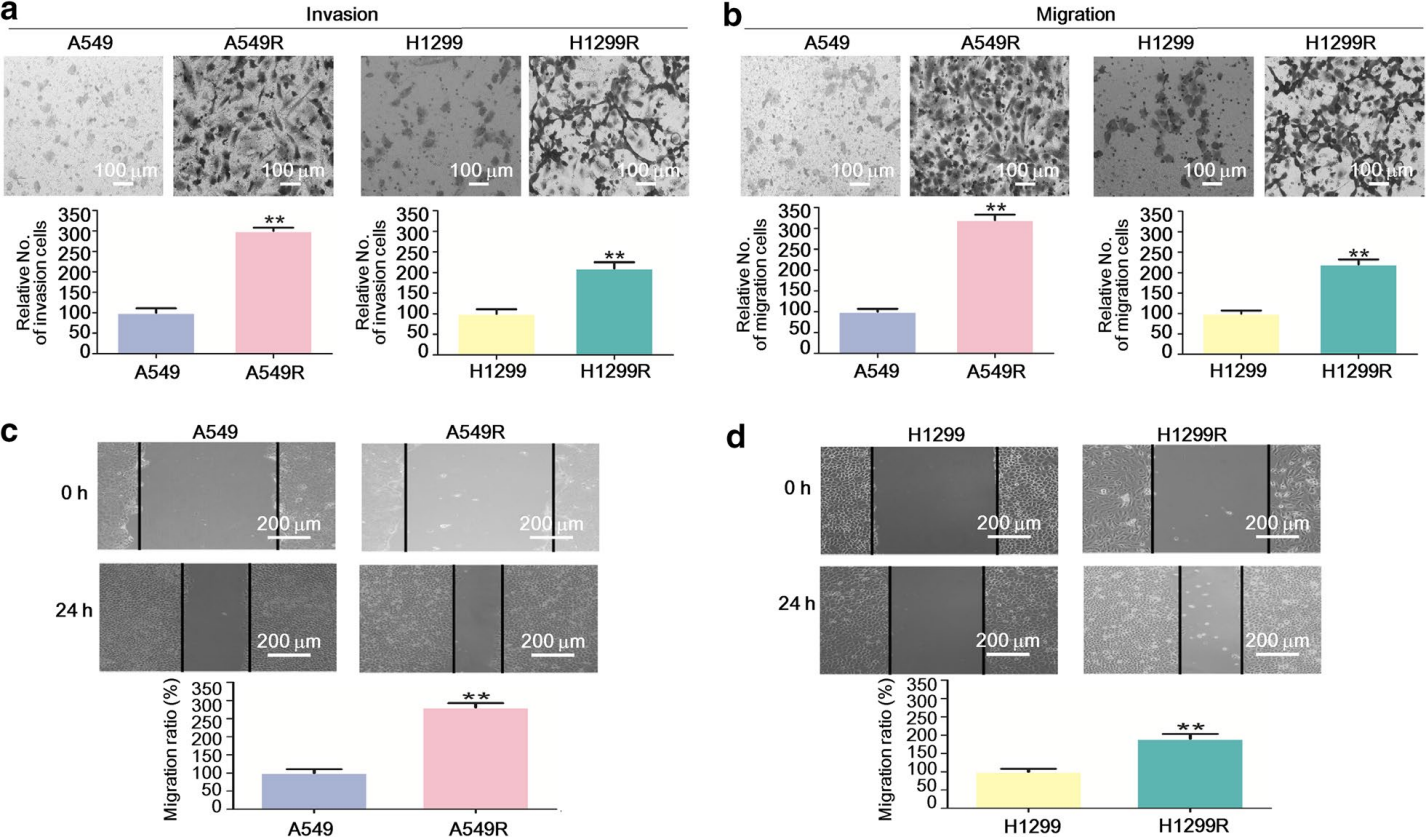
The radioresistant non-small cell lung cancer cells showed enhanced ability of invasion and migration. **a**, **b** Transwell matrigel invasion and migration assays with A549, A549R, H1299, and H1299R cells. The cells were stained with crystal violet, counted under a light microscope, and photographed at 200× magnification. The cell number was normalized to A549 or H1299 cells, respectively, and are presented as mean ± standard deviation (SD). **P < 0.01 (Student’s *t* test). **c**, **d** Wound healing assay with A549, A549R, H1299, and H1299R cells. Representative pictures are shown, and the width of the wounds was measured at 0 and 24 h after the scratch. The migration rate was normalized to A549 or H1299 cells, respectively, and is presented as mean ± SD. **P < 0.01 (Student’s *t* test)

Since EMT is closely related to metastasis [7], we next tested the ability of invasion and migration of the cells. As expected, by transwell assays, A549R and H1299R cells showed enhanced ability of invasion and migration compared with the parental cells (Fig. 3a, b). Wound healing assay by in vitro scratch method also supported the hypothesis that A549R and H1299R cells had increased migratory ability (Fig. 3c, d).

### A549R and H1299R cells had attenuated LKB1-SIK1 signaling and up-regulation of ZEB1

**Fig. 4:**
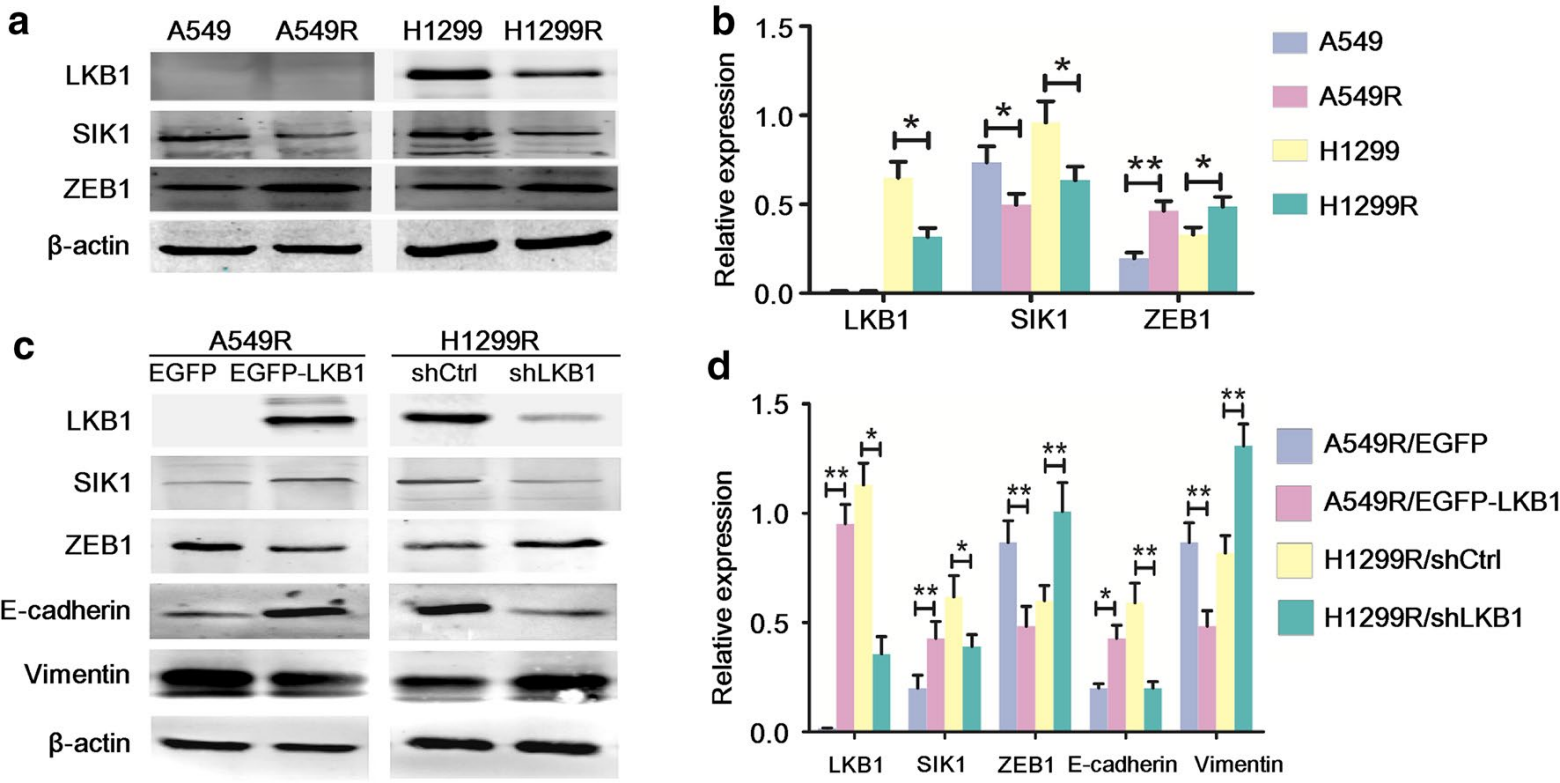
Liver kinase B1 (LKB1)-salt-inducible kinase 1 (SIK1) signaling suppressed the epithelial–mesenchymal transition phenotype of A549R and H1299R cells. **a** Western blot analysis of LKB1, SIK1, and Zinc-finger E-box-binding homeobox factor 1 (ZEB1) expression in A549, A549R, H1299, and H1299R cells. **b** The quantification of the protein levels in **a**. *P < 0.05; **P < 0.01 (Student’s *t* test). **c** Western blot analysis of LKB1, SIK1, ZEB1, E-cadherin, and vimentin expression in A549R cells transfected with enhanced green fluorescent protein (EGFP) or EGFP-LKB1 (*left panel*), and H1299R cells transfected with shCtrl or shLKB1 (*right panel*). **d** The quantification of the protein levels in **c**. *P < 0.05; **P < 0.01 (Student’s *t* test)

LKB1 is a tumor suppressor that is frequently inactivated in lung adenocarcinoma [11]. LKB1 phosphorylates and activates SIK1 to repress the expression of ZEB1 [18], and ZEB1 is a potent driver of EMT [6]. We next tested whether this pathway is dysregulated in A549R and H1299R cells. It has been reported that A549 cells have no expression of LKB1, whereas H1299 cells express wild-type LKB1 [20]. Indeed, LKB1 expression was clearly detected in H1299 cells but not in A549 cells (Fig. 4a, b). Interestingly, in H1299R cells, we found a dramatically reduced expression of LKB1 and SIK1 and increased expression of ZEB1. In A549R cells, we also observed reduced expression of SIK1 and increased expression of ZEB1, despite LKB1 not being expressed. This result suggested that dysregulation of the LKB1-SIK1-ZEB1 pathway was common in both A549R and H1299R cells, although it occurred at different levels due to different genetic backgrounds.

### LKB1-SIK1 signaling suppressed the EMT phenotype of A549R and H1299R cells

To examine LKB1’s effect on the SIK1-ZEB1 signaling and the EMT phenotype, we manipulated the expression level of LKB1 by transient transfection of vectors encoding either EGFP-LKB1 or an shRNA against LKB1. Re-expression of LKB1 in A549R cells caused increased expression of SIK1 and decreased expression of ZEB1 (Fig. 4c, d). Importantly, it also caused markedly increased expression of E-cadherin and decreased expression of vimentin, suggesting a reversal of EMT. On the other hand, knockdown of LKB1 in H1299R cells caused decreased expression of SIK1 and increased expression of ZEB1 and exacerbated the EMT phenotype. Together, our data suggest that LKB1-SIK1 signaling suppresses the EMT phenotype, and that dysregulation of the LKB1-SIK1-ZEB1 pathway may play a causative role in the EMT phenotype of A549R and H1299R cells.

### LKB1 suppressed the invasion and migration of A549R and H1299R cells

**Fig. 5:**
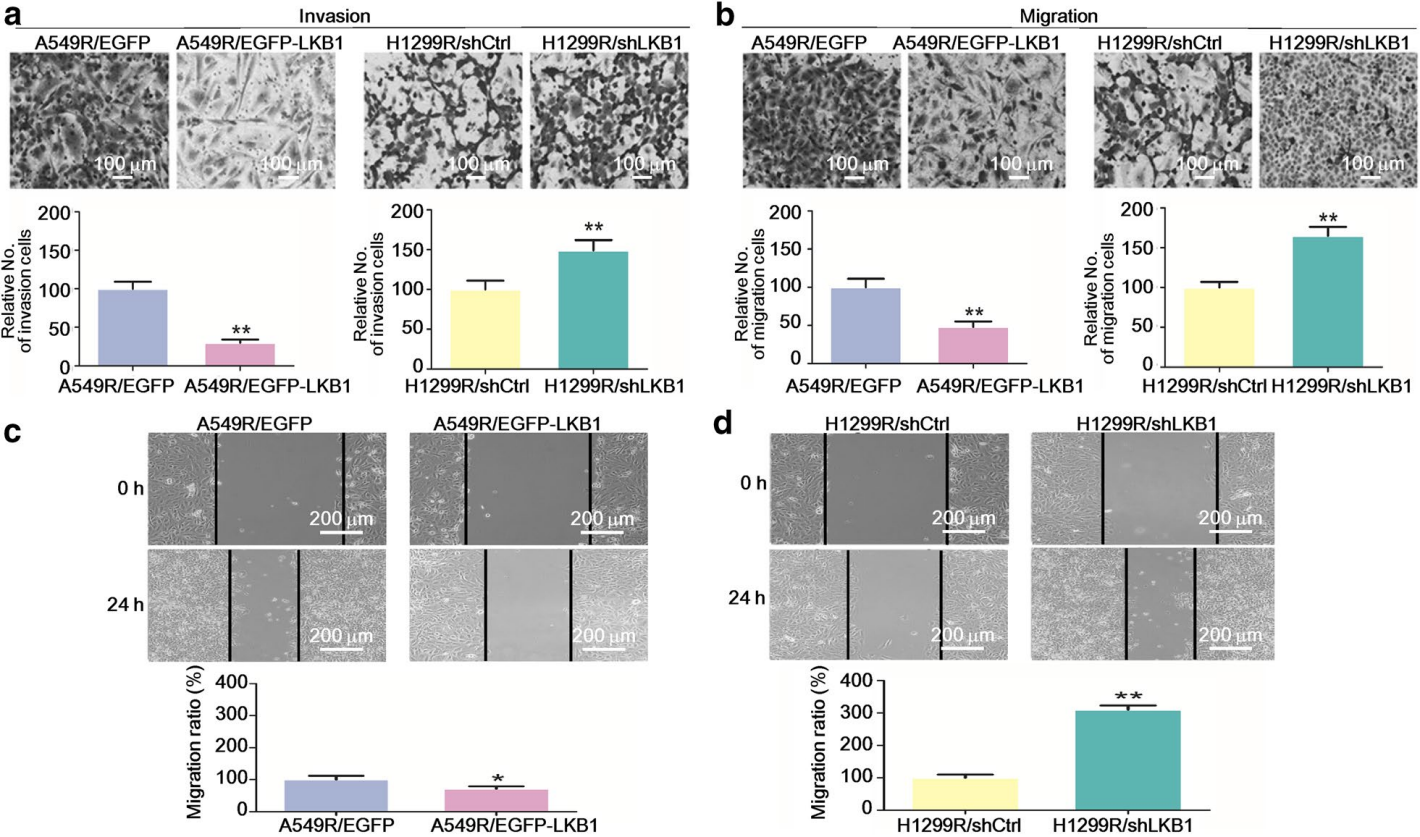
LKB1 suppressed the invasion and migration of A549R and H1299R cells. **a**, **b** Transwell matrigel invasion and migration assays with A549R cells transfected with EGFP or EGFP-LKB1, and H1299R cells transfected with shCtrl or shLKB1. **c**, **d** Wound healing assay with A549R cells transfected with EGFP or EGFP-LKB1, and H1299R cells transfected with shCtrl or shLKB1. The experimental procedure is described in Fig. 3, *P < 0.05; **P < 0.01 (Student’s *t* test)

Since LKB1 signaling was negatively associated with the EMT phenotype of A549R and H1299R cells, we then tested whether this could translate into metastatic behavior. Re-expression of LKB1 in A549R cells decreased the number of cells invaded or migrated into the lower chamber of the transwell, whereas knockdown of LKB1 in H1299R cells demonstrated the opposite effect (Fig. 5a, b). Wound healing assay also showed that LKB1 suppressed the migration of A549R and H1299R cells (Fig. 5c, d). However, we also observed that re-expression of LKB1 inhibited the proliferation of A549R cells, whereas knockdown of LKB1 promoted the proliferation of H1299R cells (data not shown). To minimize the effect of different proliferation rates, we used serum-free medium in the transwell assays; we could not, however, exclude the effects of different proliferation rates on our results. Thus, the observed suppression of invasion and migration of A549R and H1299R cells by LKB1 was likely a combined effect that included the inhibition of proliferation.

### LKB1 increased the radiosensitivity of A549 and H1299 cells

**Fig. 6:**
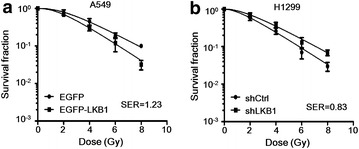
LKB1 increased the radiosensitivity of A549 and H1299 cells. The radiosensitivity was evaluated by clonogenic cell survival assay. **a**, **b** A549 cells were transfected with EGFP or EGFP-LKB1, and H1299 cells were transfected with shCtrl or shLKB1, and all were irradiated with the indicated dose. After 14 days, the colonies formed by the surviving cells were counted. The survival fraction was normalized to the non-irradiated controls and presented as mean ± SD. The survival curves were fitted to a linear-quadratic model using GraphPad Prism 5.0 software. Re-expression of LKB1 in A549 cells increased the sensitizer enhancement ratio (SER) to 1.23, whereas knockdown of LKB1 in H1299 cells decreased the SER to 0.83

Increasing evidence connects EMT and radioresistance [8]. Since LKB1-SIK1 signaling suppressed the EMT phenotype of A549R and H1299R cells as shown previously, an intriguing question is whether LKB1 could also increase the radiosensitivity. To address this question, we either re-expressed or knocked down LKB1 in A549 or H1299 cells, respectively, and subjected the cells to radiation. Indeed, as assessed by clonogenic cell survival assay, re-expression of LKB1 increased the radiosensitivity of A549 cells, whereas knockdown of LKB1 decreased the radiosensitivity of H1299 cells (Fig. 6).

## Discussion

Intrinsic and/or acquired resistance to radiotherapy has been recognized as a significant impediment to effective cancer treatment [21]. Evidence suggests that NSCLC cells that survive ionizing radiation treatment display cancer stem cell and EMT phenotypes [9]. In this study, two radioresistant NSCLC cell lines, A549R and H1299R, were established by dose-gradient irradiation of A549 and H1299 cells. As expected, A549R and H1299R cells showed increased radioresistance. Consistent with the results of other studies, our findings suggest that radiation induces the EMT phenotype with increased ability of invasion and migration.

The expression of LKB1 is frequently reduced in certain human cancers and is negatively associated with the outcome of lung cancer patients [22]. We found that the expression of LKB1 was dramatically reduced in H1299R cells compared with the parental cells. Although A549 cells did not express LKB1, the expression of SIK1 (a downstream effector of LKB1) was reduced in A549R cells. Therefore, the LKB1-SIK1 signaling was attenuated in both A549R and H1299R cells. This raises the possibility that reduced LKB1-SIK1 signaling may confer a survival advantage during and after ionizing radiation. Indeed, we showed that LKB1-SIK1 signaling was negatively associated with the EMT phenotype and increased the radiosensitivity of A549 and H1299 cells. Suppression of EMT by LKB1-SIK1 signaling is likely through repression of ZEB1, a potent driver of EMT; indeed, we observed a negative association between ZEB1 expression and EMT. Taken together, our work stresses the importance of LKB1 signaling in the radiotherapy of NSCLC, since inactivation of this pathway promotes EMT, which not only contributes to radioresistance but also increases the risk of metastasis. On the other hand, exploiting this pathway to suppress EMT might provide therapeutic benefits by increasing radiosensitivity and by reducing the risk of metastasis. EMT is also associated with resistance to chemotherapy [23]; thus, targeting the LKB1-SIK1-ZEB1 pathway to suppress EMT could have even broader therapeutic implications.

It is well known that matrix metalloproteinases (MMPs) play an important role in the invasion and metastasis of cancer cells by degrading the extracellular matrix [24]. MMPs are also closely related to EMT, since activation of the EMT process is dependent on MMPs, and overexpression of MMPs promotes EMT [24]. Interestingly, several studies reported that LKB1 can down-regulate the expression of MMP-2 and MMP-9 [25–27]. Further investigation is required to determine whether this is part of the mechanism by which LKB1 suppresses EMT.

## Conclusions

Our findings suggest that attenuated LKB1-SIK1 signaling promotes EMT and the radioresistance of NSCLC cells, which subsequently contributes to enhanced metastatic potential. Therefore, targeting the LKB1-SIK1-ZEB1 pathway to suppress EMT might provide therapeutic benefits, not only by increasing the radiosensitivity of the cancer cells but also by reducing the risk of metastasis after radiotherapy.

## Abbreviations

NSCLC: non–small cell lung cancer
LKB1: Liver kinase B1
AMPK: AMP-activated protein kinase
SIK1: salt-inducible kinase 1
ZEB1: zinc-finger E-box-binding homeobox factor 1
EMT: epithelial-mesenchymal transition
STK11: serine/threonine protein kinase 11
SD: standard deviation
CCK-8: Cell Counting Kit-8
SER: sensitizer enhancement ratio
MMPs: matrix metalloproteinases

## References

[1] Torre LA, Bray F, Siegel RL, Ferlay J, Lortet-Tieulent J, Jemal A (2015). Global cancer statistics, 2012. CA Cancer J Clin.

[2] Chen W, Zheng R, Zeng H, Zhang S (2015). The updated incidences and mortalities of major cancers in China, 2011. Chin J Cancer.

[3] Ricardi U, Badellino S, Filippi AR (2015). Stereotactic body radiotherapy for early stage lung cancer: history and updated role. Lung Cancer.

[4] Li T, Zeng ZC, Wang L, Qiu SJ, Zhou JW, Zhi XT (2011). Radiation enhances long-term metastasis potential of residual hepatocellular carcinoma in nude mice through TMPRSS4-induced epithelial–mesenchymal transition. Cancer Gene Ther.

[5] Zhang X, Li X, Zhang N, Yang Q, Moran MS (2011). Low doses ionizing radiation enhances the invasiveness of breast cancer cells by inducing epithelial–mesenchymal transition. Biochem Biophys Res Commun.

[6] Kalluri R, Weinberg RA (2009). The basics of epithelial–mesenchymal transition. J Clin Invest.

[7] Banyard J, Bielenberg DR (2015). The role of EMT and MET in cancer dissemination. Connect Tissue Res.

[8] Nantajit D, Lin D, Li JJ (2015). The network of epithelial–mesenchymal transition: potential new targets for tumor resistance. J Cancer Res Clin Oncol.

[9] Gomez-Casal R, Bhattacharya C, Ganesh N, Bailey L, Basse P, Gibson M (2013). Non-small cell lung cancer cells survived ionizing radiation treatment display cancer stem cell and epithelial–mesenchymal transition phenotypes. Mol Cancer.

[10] Shintani Y, Okimura A, Sato K, Nakagiri T, Kadota Y, Inoue M (2011). Epithelial to mesenchymal transition is a determinant of sensitivity to chemoradiotherapy in non-small cell lung cancer. Ann Thorac Surg.

[11] Matsumoto S, Iwakawa R, Takahashi K, Kohno T, Nakanishi Y, Matsuno Y (2007). Prevalence and specificity of LKB1 genetic alterations in lung cancers. Oncogene.

[12] Liu S, Miao Y, Fan C, Liu Y, Yu J, Zhang Y (2013). Clinicopathologic correlations of liver kinase B1, E-cadherin, and N-cadherin expression in non-small cell lung cancer. Appl Immunohistochem Mol Morphol.

[13] Xu P, Cai F, Liu X, Guo L (2014). LKB1 suppresses proliferation and invasion of prostate cancer through hedgehog signaling pathway. Int J Clin Exp Pathol.

[14] Ji H, Ramsey MR, Hayes DN, Fan C, McNamara K, Kozlowski P (2007). LKB1 modulates lung cancer differentiation and metastasis. Nature.

[15] Jansen M, Ten Klooster JP, Offerhaus GJ, Clevers H (2009). LKB1 and AMPK family signaling: the intimate link between cell polarity and energy metabolism. Physiol Rev.

[16] Gan RY, Li HB (2014). Recent progress on liver kinase b1 (LKB1): expression, regulation, downstream signaling and cancer suppressive function. Int J Mol Sci.

[17] Cheng H, Liu P, Wang ZC, Zou L, Santiago S, Garbitt V (2009). SIK1 couples LKB1 to p53-dependent anoikis and suppresses metastasis. Sci Signal.

[18] Eneling K, Brion L, Pinto V, Pinho MJ, Sznajder JI, Mochizuki N (2012). Salt-inducible kinase 1 regulates E-cadherin expression and intercellular junction stability. FASEB J.

[19] Bai J, Zhou Y, Chen G, Zeng J, Ding J, Tan Y (2011). Overexpression of Cullin1 is associated with poor prognosis of patients with gastric cancer. Hum Pathol.

[20] Sun LL, Zhong DS, Wu S, Bai H, Chen Z (2011). Establishment and gene expression profiling of LKB1 stable knockdown lung cancer cell line. Chin Med J (Engl).

[21] Baumann M, Krause M, Hill R (2008). Exploring the role of cancer stem cells in radioresistance. Nat Rev Cancer.

[22] Roy BC, Kohno T, Iwakawa R, Moriguchi T, Kiyono T, Morishita K (2010). Involvement of LKB1 in epithelial–mesenchymal transition (EMT) of human lung cancer cells. Lung Cancer.

[23] Iwatsuki M, Mimori K, Yokobori T, Ishi H, Beppu T, Nakamori S (2010). Epithelial–mesenchymal transition in cancer development and its clinical significance. Cancer Sci.

[24] Radisky ES, Radisky DC (2010). Matrix metalloproteinase-induced epithelial–mesenchymal transition in breast cancer. J Mammary Gland Biol Neoplasia.

[25] Zhuang ZG, Di GH, Shen ZZ, Ding J, Shao ZM (2006). Enhanced expression of LKB1 in breast cancer cells attenuates angiogenesis, invasion, and metastatic potential. Mol Cancer Res.

[26] Bardeesy N, Sinha M, Hezel AF, Signoretti S, Hathaway NA, Sharpless NE (2002). Loss of the Lkb1 tumour suppressor provokes intestinal polyposis but resistance to transformation. Nature.

[27] Ou W, Ye S, Yang W, Wang Y, Ma Q, Yu C (2012). Enhanced antitumor effect of cisplatin in human NSCLC cells by tumor suppressor LKB1. Cancer Gene Ther.

